# Genome-wide characterization of a *SRO* gene family involved in response to biotic and abiotic stresses in banana (*Musa* spp.)

**DOI:** 10.1186/s12870-019-1807-x

**Published:** 2019-05-22

**Authors:** Lu Zhang, Dengbo Zhou, Huigang Hu, Weiming Li, Yulin Hu, Jianghui Xie, Shangzhi Huang, Wei Wang

**Affiliations:** 1 State Key Laboratory of Biocontrol and Guangdong Key Laboratory of Plant Resources, School of Life Sciences, Sun Yat-sen University, Guangzhou, 510006 China; 2 Institute of Tropical Bioscience and Biotechnology, Chinese Academy of Tropical Agricultural Sciences, Haikou, 571101 China; 3 Key Laboratory of Tropical Crop Biotechnology, Ministry of Agriculture, Institute of China Southern Subtropical Crop Research, Chinese Academy of Tropical Agricultural Sciences, Zhanjiang, 524091 China; 4 School of Life Sciences, Sun Yat-sen University, Guangzhou, 510275 Guangdong China

**Keywords:** *SRO* family, Banana, Induced expression, Abiotic stress, Biotic stress, hormone treatment, Protein interaction

## Abstract

**Background:**

Banana (*Musa* spp.) is one of the world’s most important fruits and its production is largely limited by diverse stress conditions. SROs (SIMILAR TO RCD-ONE) have important functions in abiotic stress resistance and development of plants. They contain a catalytic core of the poly(ADP-ribose) polymerase (PARP) domain and a C-terminal RST (RCD-SRO-TAF4) domain. In addition, partial SROs also include an N-terminal WWE domain. Although a few of SROs have been characterized in some model plants, little is known about their functions in banana, especially in response to biotic stress.

**Results:**

Six *MaSRO* genes in banana genome were identified using the PARP and RST models as a query. Phylogenetic analysis showed that 77 SROs from 15 species were divided into two structurally distinct groups. The SROs in the group I possessed three central regions of the WWE, PARP and RST domains. The WWE domain was lacking in the group II SROs. In the selected monocots, only MaSROs of banana were present in the group II. Most of *MaSROs* expressed in more than one banana tissue. The stress- and hormone-related cis-regulatory elements (CREs) in the promoter regions of *MaSROs* supported differential transcripts of *MaSROs* in banana roots treated with abiotic and biotic stresses. Moreover, expression profiles of *MaSROs* in the group I were clearly distinct with those observed in the group II after hormone treatment. Notably, the expression of *MaSRO4* was significantly upregulated by the multiple stresses and hormones. The MaSRO4 protein could directly interact with MaNAC6 and MaMYB4, and the PARP domain was required for the protein-protein interaction.

**Conclusions:**

Six MaSROs in banana genome were divided into two main groups based on the characteristics of conserved domains. Comprehensive expression analysis indicated that *MaSROs* had positive responses to biotic and abiotic stresses via a complex interaction network with hormones. MaSRO4 could interact directly with MaNAC6 and MaMYB4 through the PARP domain to regulate downstream signaling pathway.

**Electronic supplementary material:**

The online version of this article (10.1186/s12870-019-1807-x) contains supplementary material, which is available to authorized users.

## Background

Plants are persistently challenged by numerous biotic and abiotic environmental stresses. Multiple stress factors result in an extensive loss of agricultural production worldwide [1]. During evolution, plants have developed sophisticated mechanisms to protect themselves against multiple stresses. Several molecules, such as transcription factors (TFs), reactive oxygen species (ROS), cytosolic Ca^2+^ and kinases, are involved in different stress signaling pathways [2, 3]. Additionally, hormone signaling pathways regulated by salicylic acid (SA), jasmonic acid (JA), abscisic acid (ABA) and ethylene also play key roles in crosstalk between biotic and abiotic stresses [4–6].

A SIMILAR TO RCD ONE (SRO) family is a group of plant-specific proteins, which participate in abiotic stress and developmental processes [7]. In *Arabidopsis thaliana*, a radical-induced cell death 1 (AtRCD1) was originally discovered according to the recovery of oxidative stress response defects in yeast mutant [8]. Subsequently, homologs (AtSRO1–5) of AtRCD1 were also identified and divided into two structural types [7]. *AtRCD1* and *AtSRO1* in the type I contain an N-terminal WWE domain (PS50918), a poly(ADP-ribose) polymerase (PARP) domain (PS51059), and a C-terminal RCD1-SRO-TAF4 (RST) domain (PF12174) [9]. The type II includes AtSRO2, 3, 4, and 5 lacking the WWE domain [7]. Previous studies demonstrated that the RST domain was specifically involved in plant SRO and TAF4 proteins, while the WWE-PARP domain widely existed in various organisms [10, 11]. The WWE domain might be required for protein-protein interactions by forming a globular structure [12]. The RST domain is essential for the SRO interaction with different TFs [7, 9].

Until now, limited knowledge of SROs was obtained from *Arabidopsis* and rice. A loss-of-function mutation of *AtRCD1* resulted in the increased sensitivity to the abiotic stress responses, the aberrant leaf and rosette morphology, and the altered hormone responses [13–16]. AtRCD1 was also involved in salt stress by interacting with SOS1, a plasma membrane Na^+^/H^+^ antiporter [17]. However, little work had been done on *AtSRO2*, *3*, *4* and *5*. *AtSRO2* was upregulated in response to light treatment. *AtSRO3* was significantly downregulated under light stress, but induced by salt stress and ozone [7]. The mutated *AtSRO5* plants were more sensitive to H_2_O_2_-mediated oxidative stress and salt stress [18]. In addition, a rice SRO protein OsSRO1c had dual roles in improving drought and oxidative stress tolerance by the interaction with NAC1 and zinc finger TFs [19, 20]. The wheat SRO could alleviate the oxidative stress under salt treatment by modulating redox homeostasis [212]. Likewise, overexpression of apple *RCD1* in transgenic apple calli and *Arabidopsis* plants enhanced the plant resistance to salt stress [22]. However, whether members of the *SRO* gene family also participate in the regulation of biotic stress is still an open question.

Although some functional studies on this family have been carried out in the model plants, very little is known in the non-model plants. Banana is vital for food security in many tropical and subtropical countries. The vegetative propagation of banana commercial cultivars resulted in the susceptibility to pests and diseases due to the narrow genetic background [23]. For example, fusarium wilt, caused by *Fusarium oxysporum* f. sp. *cubense* (*Foc*), is one of the most destructive diseases, which can cause leaf wilt and death of the whole plant. Especially, a strain of *Foc* called tropical race 4 (*Foc* TR4) has overcome more than 80% of global banana and plantain [24]. Moreover, adverse environmental factors such as drought and low temperature also restrict the industrial development of global banana [25, 26]. To understand *MaSRO* function in response to abotic and biotic stresses, we first identified and characterized the *SRO* gene family of banana genome. Expression patterns of *MaSROs* were detected in response to six abiotic stresses (cold, osmotic, salinity, ultraviolet, heat and, wounding), one biotic stress (*Foc* TR4 inoculation), and four hormone treatments (ABA, SA, GA, and ethylene). By contrast, *MaSRO4* was upregulated by multiple stress responses and interacted with MaNAC6 and MaMYB4 by the PARP domain.

## Results

### Identification and classification of MaSROs

**Fig. 1:**
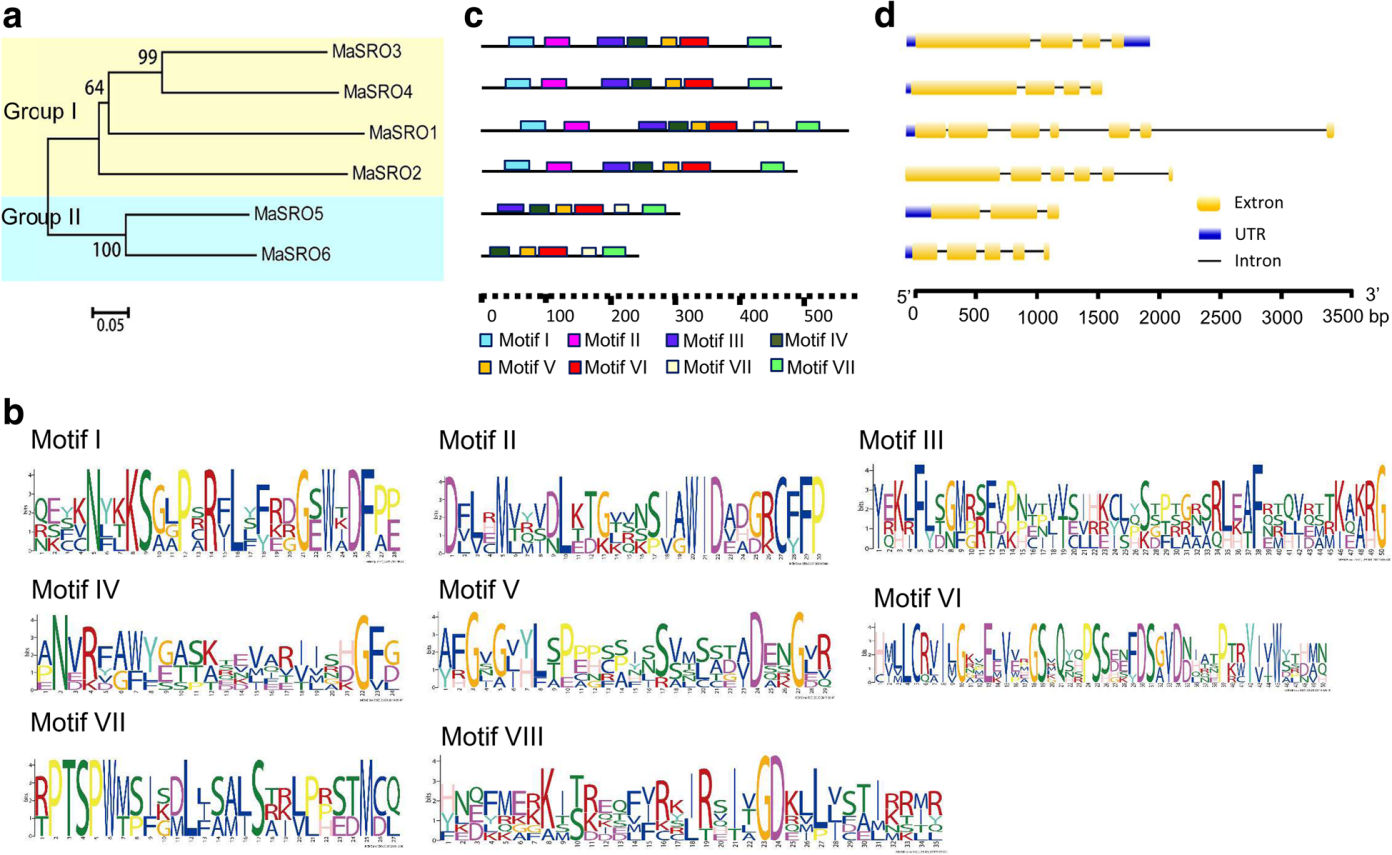
Analysis of evolutionary relationship, protein domains and gene structures of the banana *SRO* gene family. **a** Six MaSROs clustered into two groups in an unrooted ML tree constructed by MEGA7.0. The group I contained MaSRO1, 2, 3 and 4. MaSRO5 and MaSRO6 formed the group II. **b** Seven conserved motifs of MaSROs were generated by the online MEME tool. The overall height of the stack represented the level of sequence conservation. Heights of residues within a stack indicated the frequency of each residue at the indicated position. **c** The positions of identified seven motifs were schematically presented in MaSROs. Different motifs were displayed using the different colored boxes. The lengths of proteins and motifs were estimated using the scale at the bottom. **d** The intron/exon structures of *MaSROs* were analyzed by comparing the genomic and cDNA sequences. Yellow and black boxes represented the exon and intron regions, respectively. Blue boxes indicated the untranslated regions (UTRs). The sizes of exons and introns were measured using the scale at the bottom

After searching the entire genome of banana using the PARP and RST models, six *MaSROs* were obtained and named sequentially from *MaSRO1* to *MaSRO6* according to the description of Jaspers et al. (2010) [7]. A Maximum-Likelihood (ML) phylogenetic tree was produced using the full-length protein sequences of MaSROs. Six MaSROs clustered into two distinct groups (Fig. 1a). The group I contained four members from MaSRO1 to MaSRO4 with the WWE, PARP and RST domains, while MaSRO5 and MaSRO6 lacking the WWE domain belonged to the group II. To further identify other conserved domains, we analyzed all amino-acid (AA) sequences of MaSROs against the MEME tool [22]. In total, seven conserved motifs were listed in Additional file 1. MaSRO5 and MaSRO6 evidently lacked the internal motif I and motif VI, whereas motif VI was only found in MaSRO2 (Fig. 1b,c). Two highly conserved Motifs V and VII in the C-termini of MaSROs harbored the typical PARP and RST domains, respectively. Motif I within the WWE domain mainly located in the N-termini. The structure distribution of motifs supported the grouping results (Fig. 1a).

To determine the genomic structures of *MaSROs*, each DNA sequence was used to search the banana-genome database. Distributions of *MaSROs* on banana 12 chromosomes seemed to be uneven (Additional file 2). Chromosomes 4 and 5 contained two *MaSROs*, respectively. *MaSRO2* and *MaSRO4* located on chromosome 7 and 9, respectively. Exon/intron structures of *MaSROs* (such as between *MaSRO3* and *MaSRO4*, between *MaSRO1* and *MaSRO2*) were conserved within the same subfamily, except for *MaSRO6* and *MaSRO*7 (Fig. 1d).

We also predicted the chemical and physical characters of each MaSRO protein. The lengths of MaSROs range from 240 to 490 AAs, and the values of GRAVY change from − 0.202 to − 0.435. Based on an instability index, most of MaSROs belong to unstable proteins. MaSROs have obvious changes in isoelectric point from 6.33 to 9.69 and in molecular weight from 27.023 kDa to 66.147 kDa. The subcellular localizations of MaSRO1, 2, 3 and 5 were predicted in chloroplast or nucleus, whereas MaSRO4 and MaSRO 6 located in nucleus shown in Additional file 2.

### Phylogenetic analysis of the *SRO* gene family

**Fig. 2:**
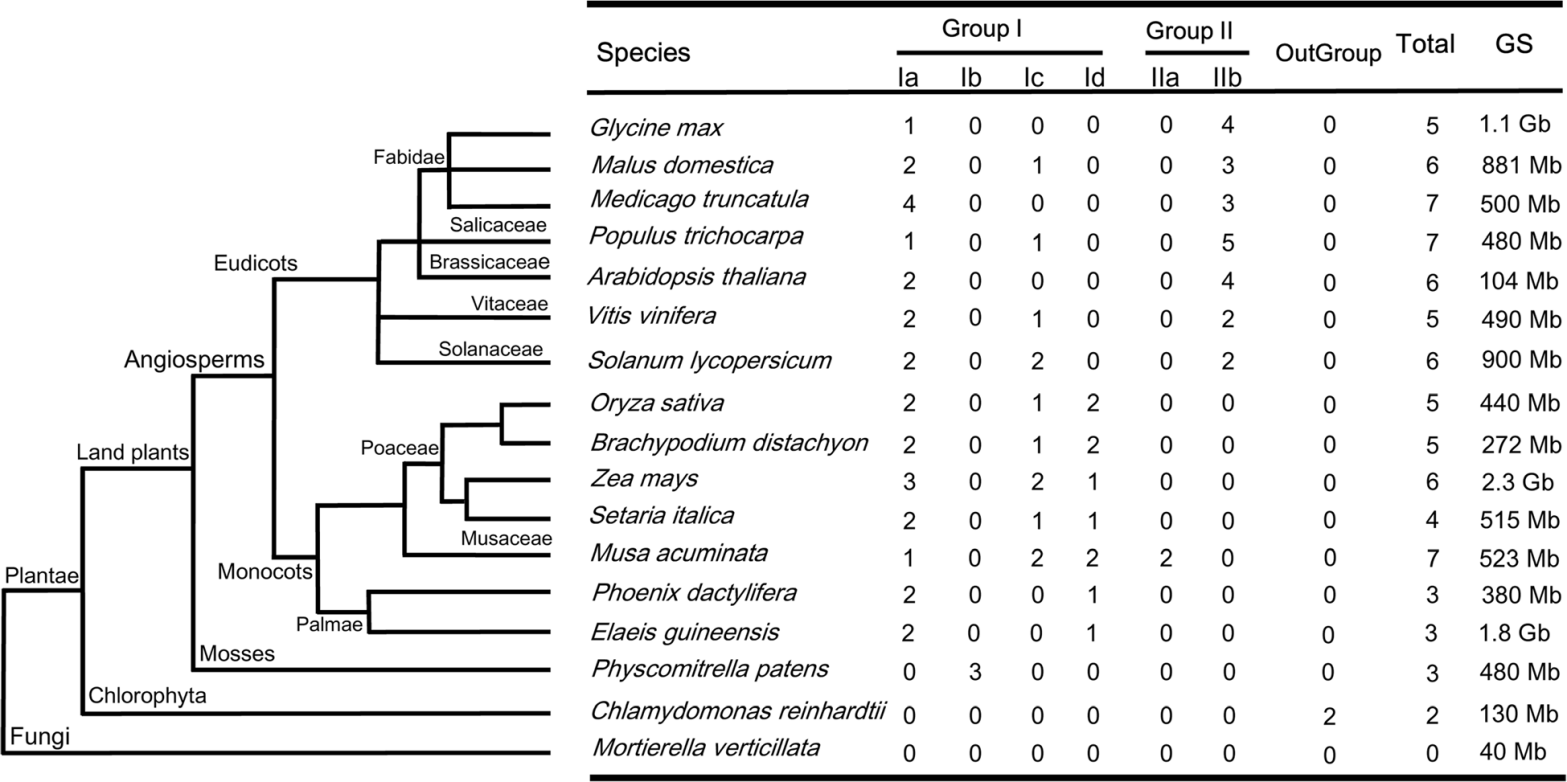
Distribution of the SROs in Plantae. The left graph indicated the categories of species. The nodes of tree represented the evolutionary relationship. The number and classification of SROs in each species were described in the right diagram. GS: genome size

To investigate the evolutionary relationship among plant SRO proteins, we identified 80 SROs in 16 genome-sequenced species including *A. thaliana* (6), *Zea mays* (6), *Populus trichocarpa* (7), *Musa acuminata* (6), *Solanum lycopersicum* (6), *Vitis vinifera* (5), *Physcomitrella patens* (3), *O. sativa* (5), *Medicago truncatula* (7), *Malus domestica* (6), *Brachypodium distachyon* (5), *Setaria italica* (4), *Chlamydomonas reinhardtii* (2), *Glycine max* (5), *Elaeis guineensis* (3), and *Phoenix dactylifera* (3) in Additional file 3. The selected plants represented species within the division of angiospermae and Bryophyta (Fig. 2). The numbers of SROs in different plant species showed a gradual increase from algae to flowering plants along with the increase of organism complexity [27]. Most plants owned 5–7 SRO members in their genomes. *E. guineensis*, *P. patens* and *P. dactylifera* contained three SROs, while only two SRO homologs were identified in the *C. reinhardtii* genome. No SRO homologs were observed in a eukaryotic microalga *Coccomyxa subellipsoidea* and a unicellular green alga *Ostreococcus lucimarinus*. Likewise, we did not also found SRO homologs in the photosynthetic and/or eukaryotic microorganisms. Loss of ancestral *SROs* in specific lineages supports the evolutionary diversification through extensive expansion during plant evolution.

**Fig. 3:**
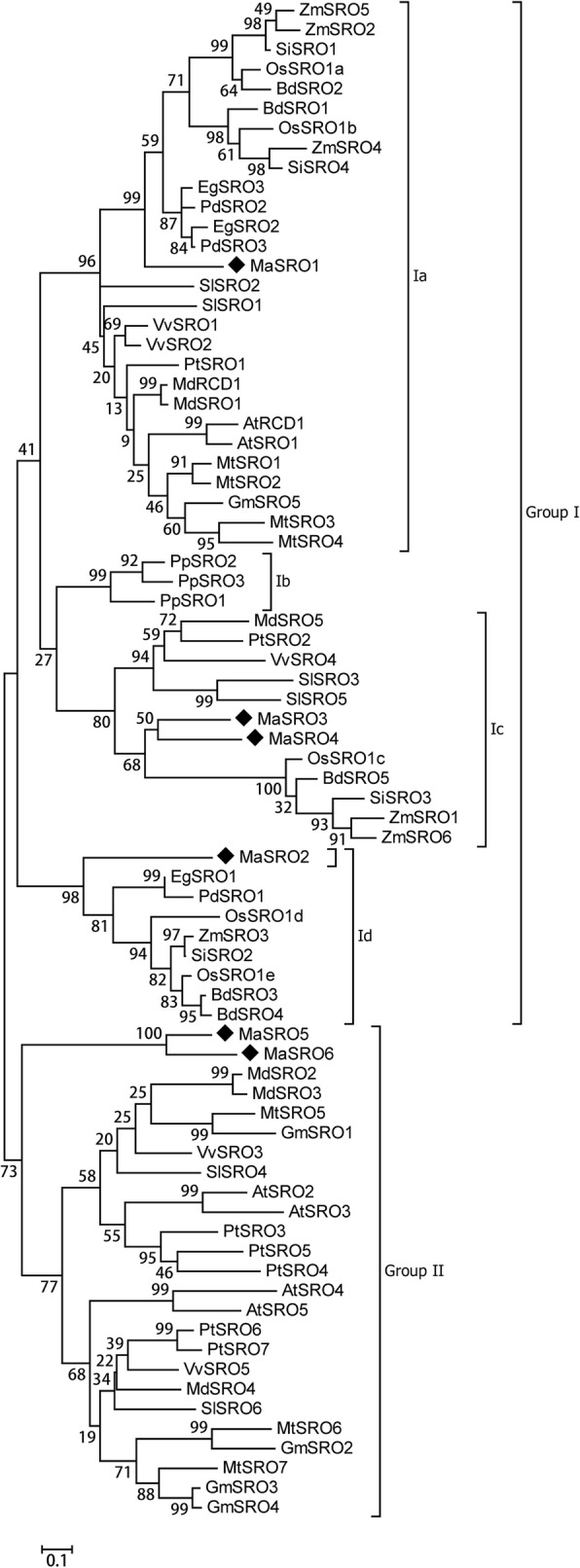
Phylogenetic analysis of the SROs from different plants. A total of 77 SROs were identified from 15 representative plant species. The ML phylogenetic tree was constructed using the MEGA7 software based on the full-length protein sequences of SROs. Numbers on branches were bootstrap values calculated from 1000 replicates. These SROs were clustered into two main groups and five subgroups. The scale bar indicated AA substitutions per position

Based on the full-length protein sequences of SROs (Additional file 4), a ML phylogenetic tree was generated using bootstrap analysis (1000 replicates) (Additional file 5). Deviation seemingly happened from *C. reinhardtii* with a separate monophyletic clade, thereby confounding the tree topology. Hence, we removed two CrSROs from the final phylogenetic tree. A total of 77 SRO members from 15 species were clustered into the group I and II (Fig. 3). Based on the branch value (> 75) [28], the group I was further classified into four subgroups (Ia, Ib, Ic, and Id) [7] . AtRCD1 and AtSRO1 formed the subgroup Ia with the SRO homologs from all selected species except for *P. patens*, where an obvious divergence branch was produced between monocots and eudicots (Fig. 2). Three PpSROs of *P. patens* independently consisted of subgroup Ib. Only monocots in our selected species were involved in subgroup Id. The group II can be classified into one group. Besides MaSRO5 and MaSRO6 from banana, no SRO homologs of other monocots belonged to the subgroup II.

### Characteristics of conserved domains in plant SROs

Based on the domain architecture, those SROs in the group I had a central region of the WWE, PARP and RST domains. No WWE domain was found in the SRO members of group II. Compared with compositions of AAs in three domains, an obvious variation was observed in the WWE domain (Additional file 6). The RST domain was most conserved in monocots. There are a strong conservation of aliphatic AAs in the N-terminus, a strictly conserved tyrosine (Y) in the middle of RST domain, and two glycine (G) and aspartic acid (D) residues in the C-terminus. In addition, seven conserved motifs were used to demonstrate the domain architecture of plant SROs from 15 species (Additional file 7). The basic PARP domain containing motif I and motif VII was found in all SROs, suggesting that this domain was important for basic function of SROs. It was supported that the phylogenetic trees constructed using the conserved PARP domain and full-length protein sequences were similar, although a difference was generated in the definition of subgroup (Additional file 8). The motif II within the variable WWE region was shown in the group I, except for PpSRO1 from *P. patens*. Especially, most of members in the same subgroup shared one or more motifs outside the PARP and RST domains, further supporting the subgroup definition (Additional file 7).

### Expression profiles of *MaSROs* in different banana tissues

A quantitative real-time PCR (qRT-PCR) was performed to investigate expression patterns of *MaSROs* in banana (*Musa* AAA, cv. Williams) roots, leaves, stems, and fruits (Additional file 9). The PCR product of each *MaSRO* was confirmed by sequencing. Transcripts of *MaSROs* could be detected in the all selected four tissues, but low expression levels were observed in fruits. Remarkably, *MaSRO2* exhibited relatively high expression levels in roots, while low transcript accumulation was detected in banana stems and leaves. *MaSRO1*, *3* and *4* clustering into the same group showed a preferential expression in stems. *MaSRO5* had a constitutive expression in different organs. Our results suggest that *MaSROs* might play key roles in multiple biological processes during growth of banana plants.

### Expression characteristics of *MaSROs* under diverse abiotic stresses

**Fig. 4:**
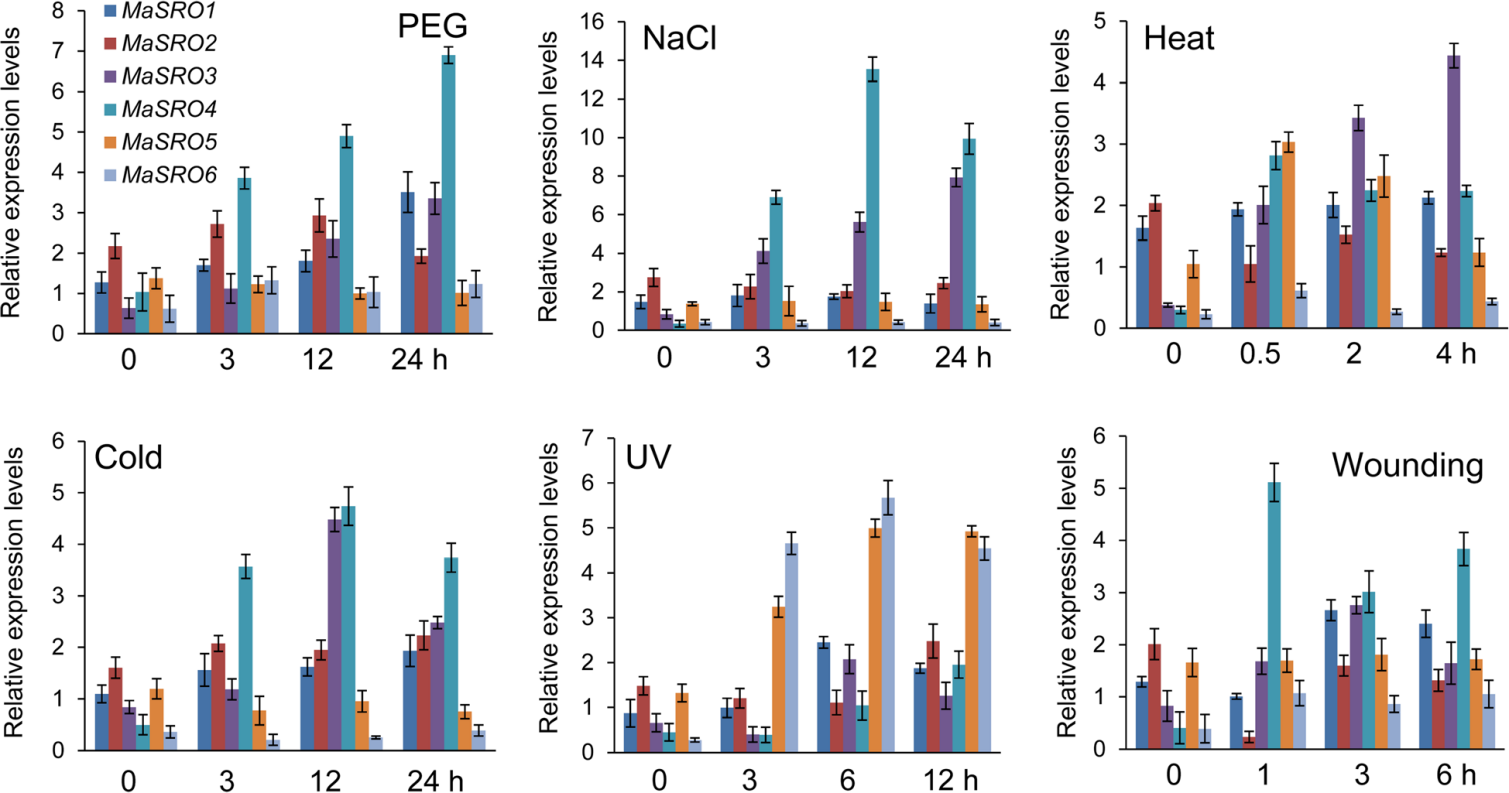
qRT-PCR expression analysis for six *MaSROs* in banana roots under diverse abiotic stresses. The treated banana roots were harvested at the indicated time points. Data indicated relative expression levels (means ± SE) from three independent biological replica (three RNA extractions; *n* = 3). Used primers specific for *MaSROs* were listed in Additional file 10

To identify potential functions of *MaSROs* in response to different abiotic stresses, their transcript profiles were assayed under polyethylene glycol (PEG), salt, cold, heat, ultraviolet (UV) and wounding treatments (Fig. 4). The PEG treatment was used to simulate osmotic stress. At the initial stage, the expression levels of *MaSROs* were obviously upregulated except for *MaSRO5*. Especially, the transcript levels of *MaSRO1, 3* and *4* gradually increased under PEG treatment until 24 h. No significant difference was found in the transcription accumulation of *MaSRO5* among the indicated time points*.* Under salt treatment, *MaSRO3* and *MaSRO4* reached an expression peak at 24 h and 12 h, respectively, while other members were downregulated or showed no significant changes. By contrast, expression of *MaSRO1, 3, 4* and *5* could be induced by heat treatment. The increase of *MaSRO3* and *MaSRO4* ranged from 5.41- to 11.76-folds and the enhancement of *MaSRO1* and *MaSRO5* were between 1.18- and 3.01-folds. Under cold stress, an obvious upregulation was observed in the expression levels of *MaSRO1*, *2*, *3* and *4*, but both *MaSRO5* and *MaSRO6* were down-regulated. Notably, the expression levels of *MaSRO5* and *MaSRO6* were induced at the early stage under UV stress, while the transcripts of other members were repressed. The wounding treatment induced rapidly the up-regulation of *MaSRO3* and *MaSRO4*. Opposite results were detected in the expression patterns of *MaSRO2*. Hence, transcripts of these *MaSROs* were responsive to most of the applied stress treatments. Especially, *MaSRO4* exhibited significant changes under multiple stress treatments, suggesting that it may own a unique role in stress responsiveness.

### Expression profiles of *MaSROs* in response to diverse hormone treatments

**Fig. 5:**
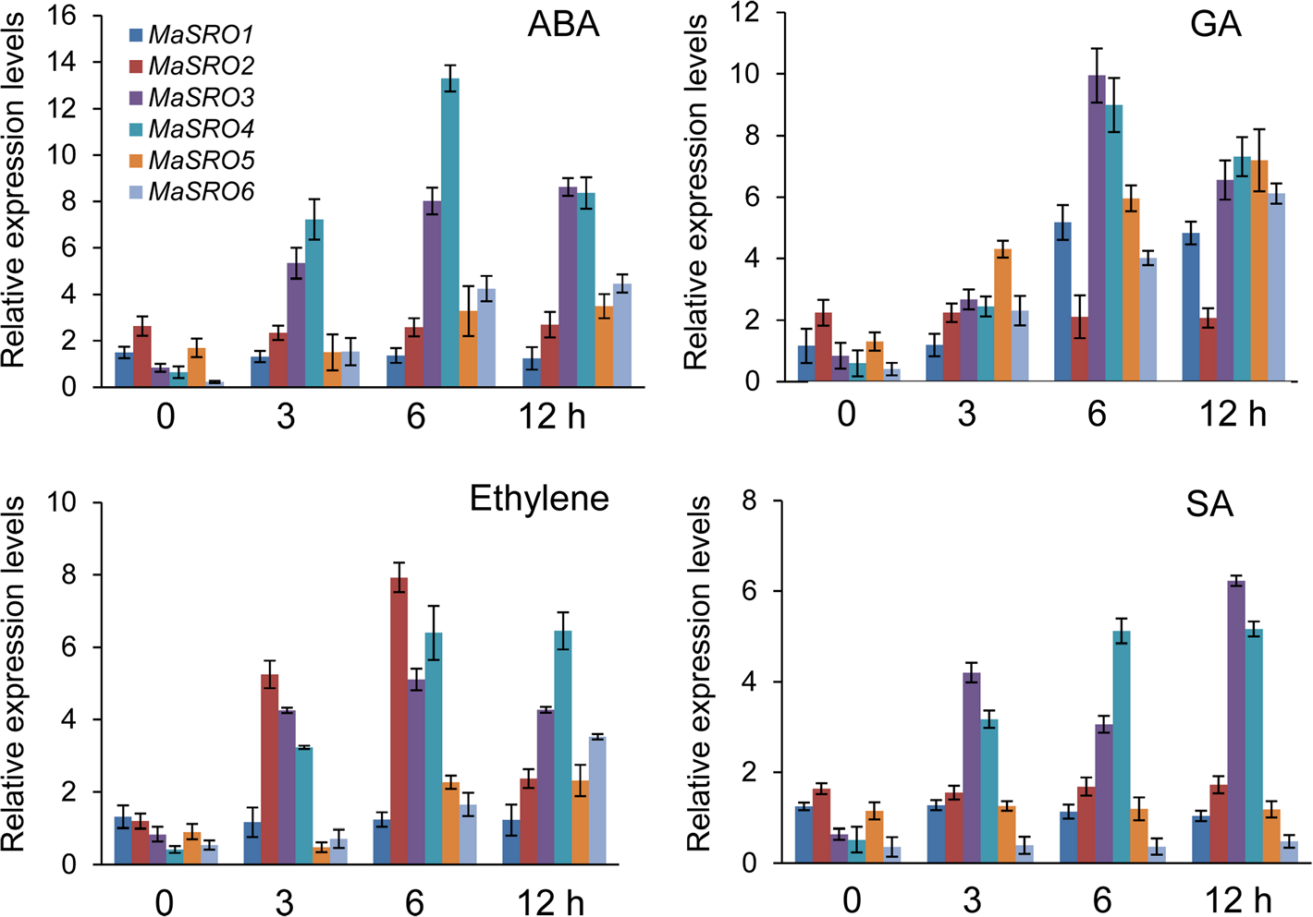
Expression profiles of *MaSROs* under various hormone treatments by qRT-PCR. The treated banana roots were harvested at the indicated time points. Data indicate relative expression levels (means ± SE) from three independent biological replica (three RNA extractions; n = 3). Used primers specific for *MaSROs* were listed in Additional file 10

Previous evidences indicated that different hormones play important roles in stress signal transduction and cell responses [4–6]. Here, we investigated the expression profiles of *MaSROs* in response to ABA, GA, ethylene, and SA treatments (Fig. 5). The highest numbers of *MaSROs* were induced by ABA treatment, followed by GA or ethylene treatment. Interestingly, SA treatment cannot significantly upregulated the expression levels of *MaSRO*s, except for *MaSRO3* and *MaSRO4. MaSRO5* and *MaSRO6* were more sensitive to ABA or GA treatment. Compared with abiotic stresses, *MaSROs* in the same subgroup showed analogous responses to exogenous hormones. For example, *MaSRO2* from the subgroup Id displayed similar expression patterns after GA or ethylene treatment. We also found that the induced transcript profiles of *MaSROs* in the group II were clearly different from those observed in the group I. *MaSRO2*, *3* and *4* in the group I showed an expression peak at the indicated time points after GA or ethylene treatment., *MaSRO5* and *MaSRO6* in the group II showed a continual transcript increase throughout the detected time points.

### Expression profiles of *MaSROs* in banana roots inoculated with *Foc* TR4

**Fig. 6:**
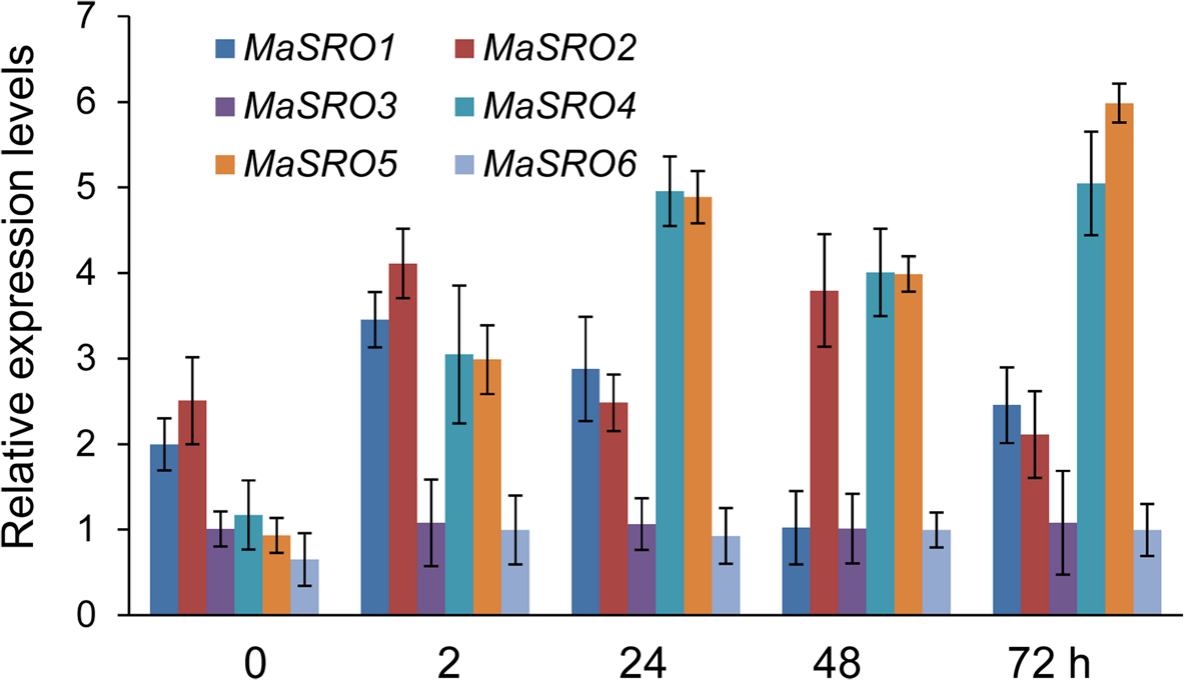
qRT-PCR analysis of *MaSROs* in banana roots inoculated with *Foc* TR4. The samples were harvested at 0, 2, 24, 48 and 72 h after *Foc* TR4 inoculation. Data indicate relative expression levels (means ± SE) from three independent biological replica (three RNA extractions; n = 3). Used primers specific for *MaSROs* were listed in Additional file 10

To investigate whether *MaSROs* are required for biotic stress, their transcription levels were analyzed in banana roots after inoculation with *Foc* TR4 (Fig. 6). Except for *MaSRO3* and *MaSRO6*, most of *MaSROs* increased by more than 2-fold at 2 h post inoculation (hpi) in comparison with the initial stage. During *Foc* TR4 infection, the expression of *MaSRO2* displayed a sharp increase, reached two peaks at 2 hpi and 48 hpi and then decreased in the following time points. *MaSRO4* and *MaSRO5* showed biphasic expression patterns with upregulation at 24 hpi and downregulation at 48 hpi. The expression peaks of *MaSRO4* and *MaSRO5* were detected at 72 hpi. Both *MaSRO3* and *MaSRO6* demonstrated low or moderate levels in consecutive expression windows.

### Stress- and hormone-induced cis-regulatory element (CRE) analysis

**Fig. 7:**
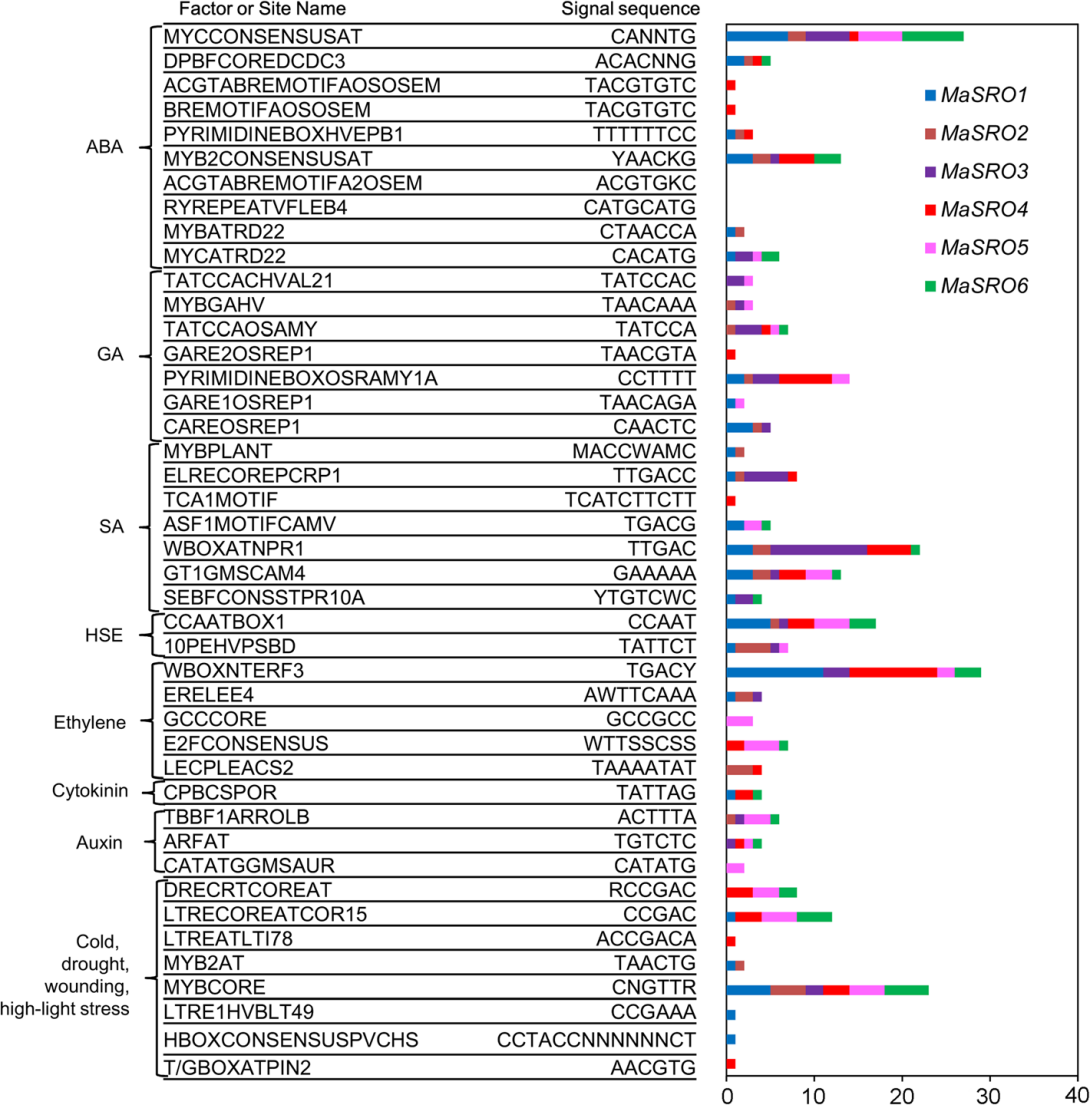
CRE analysis of *MaSROs* in 1.5-kbp upstream region of translation start codon. The left graph presented the site names and sequences of CREs involved in the putative signaling pathways. The colored columns showed the number of the identified CREs in the promoter region of each *MaSRO*

Transcriptional control of gene expression depends on CREs in the promoter region. In our study, the 1.5-kb upstream regions of the translational start sites of *MaSROs* were used for identification of CREs. Some stress- and hormone -related CREs were found according to their potential responsive functions (Fig. 7). More than one CRE were detected in the promoter region of each *MaSRO*. By contrast, most frequent CREs were identified, such as MYCCONSENSUSAT (CANNTG, ABA response factor binding site), GT1GMSCAM4 (GAAAAA, ethylene response factor binding site), WBOXNTERF3 (TGACY, responsible for pathogen- and salt-induced expression), W-Box within ELRECOREPCRP1 (TTGAC, recognized specifically by SA-induced WRKY DNA-binding proteins), ASF1 MOTIFCAMV binding site (TGACG, involved in transcriptional activation by auxin and SA treatments), low-temperature responsive elements (CCGAAA, ACCGACA and CCGAC), and auxin response factor binding sites (TGTCTC). There was an obvious difference in the type and abundance of CREs in six *MaSROs* promoters analyzed (Fig. 7). The HSE, W-box, and CBF/DREB1 elements were scattered in the promoter regions of all *MaSROs*. The promoter of *MaSRO4* contained the most diverse collection of putative CREs (a total of 25 CREs, such as CBF/DREB1, LTR, HSE, GARE, SEBF, ABRE, W-box and ERF3, etc). Some hormone response elements including TACGTGTC (ABA), TAACGTA (GA), TCATCTTCTT (SA), and AACGTG (JA) were specifically detected in the *MaSRO4* promoter. In the *MaSRO1* promoter, GA response elements such as TATCCAC, TAACAAA and TATCCA were lacking when compared to *MaSRO3* and *MaSRO4*. Only a small type of potential CREs were identified in the promoters of *MaSRO1*, *MaSRO2* and *MaSRO6*. The GA and SA response elements were absent in the *MaSRO6* promoter. By combining expression patterns of *MaSROs* with CER analysis, we found that *MaSROs* in response to multiple stimuli had a positive correlation with the types and numbers of CREs.

### Identification of interaction proteins with MaSRO4

**Fig. 8:**
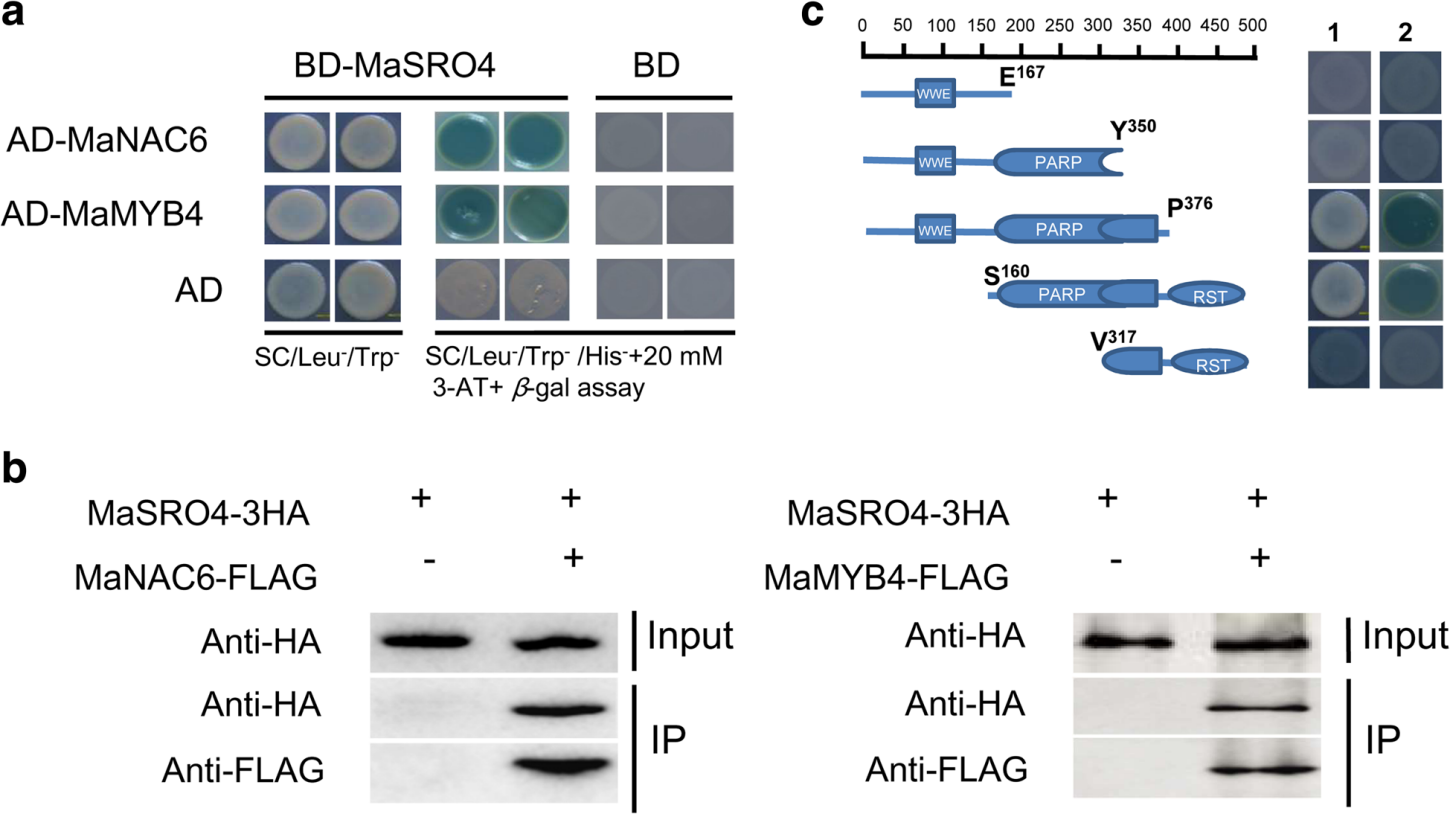
MaSRO4-interacting proteins identified by the yeast two-hybrid and Co-IP assays. **a** Yeast two-hybrid assay of MaSRO4 interacting with MaMYB4 or MaNAC6. AD-MaMYB4 or -MaNAC6 co-transformed with BD empty vector was used as negative controls. The adjacent two clones showed different colonies of each interaction test. **b** Co-IP assay of MaMYB4 or MaNAC6 interacting with MaSRO4. Genes encoding MaSRO4 tagged with 3HA (MaSRO5-3HA) and MaMYB4 or MaNAC6 tagged with FLAG (MaMYB4- or MaNAC6-FLAG) were co-expressed in *Arabidopsis* protoplasts. Protein extracts (Input) were immunoprecipitated with anti-FLAG resin. Immunoblots were developed with anti-HA antibody to detect MaSRO4 and with anti-FLAG antibody to detect MaMYB4 and MaNAC6. **c** Interactions between MaMYB4 and MaSRO4 truncated fragments using the yeast two-hybrid system. Various deletions constructs of MaSRO4 were prepared for domain-domain interaction. The left graphic presented the schematic domains of MaSRO4 and deletion derivatives. Numbers above each truncation indicated the AA coordinates of MaSRO4. The right graph showed the yeast growth (No. 1) and the *β-*galactosidase assay (No. 2)

Earlier studies indicated that AtRCD1 and OsSRO1c could interact with numerous TFs (DREB2B, AP2/ERF, MYB, bZIP and NAC families) to activate stress signaling pathways [7, 9, 19, 20]. Because expression of *MaSRO4* was significantly induced by multiple stress treatments, we further analyzed its interaction proteins to predict the stress-related regulation mechanism. Domain analysis uncovered the presence of a complete WWE-PARP-RST structure in MaSRO4, suggesting that the protein may also interact with homology TFs of rice or *Arabidopsis*. Here, yeast two-hybrid assay and immunoprecipitation (Co-IP) were performed to identify interaction proteins of MaSRO4. A MaSRO4-BD construct was used as a bait protein, and the selected banana TFs were constructed into the prey vector. By the growth of yeast on selection medium (lacking Leu, Trp, His, and Ade) and *α*-galactosidase assay, the full-length MaSRO4 protein can directly interact with MaNAC6 and MaMYB4 (Fig. 8a). Furthermore, we co-expressed *MaSRO4*-*3HA* and *TF-FLAG* in *Arabidopsis* protoplasts. Anti-FLAG resin was used for Co-IPs (Fig. 8b). Western blot analysis with anti-3HA antibody showed that the precipitated fraction contained MaSRO4-3HA. Hence, MaSRO4-3HA could be immuno-precipitated by MaNAC6−/MaMYB4-FLAG.

To understand whether the different domains of MaSRO4 are necessary for its interaction with MaMYB4, different N- and C-terminal truncations were generated by decreasing the length of MaSRO4. MaSRO4 lacking the WWE domain did not substantially affect the interaction with MaMYB4 in yeast two-hybrid system (Fig. 8c), suggesting that the WWE domain was not necessary for protein interaction. However, it was not possible to test the RST domain alone in this system, because a C-terminal construct lacking both the WWE and PARP domains strongly auto-activated the expression of the reporter gene in yeast. Deletions of the PARP domain resulted in loss of interaction with MaMYB4, indicating that the domain was required for MaSRO4 function.

## Discussion

Although SRO homologs widely exist in the kingdom of Plantae, only a few members from rice and *Arabidopsis* had been characterized to regulate plant development and improve plant’s resistance to abiotic stress [13–20]. In our study, six *MaSROs* were identified in banana genome. The phylogenetic analysis demonstrated that SRO orthologs from the 15 species clustered in the two main groups based on the full-length protein sequences (Fig. 3) and the PARP domain (Additional file 8) [7, 21]. The group II was considered previously as the eudicot group of flowering plants [7], but MaSRO5 and MaSRO6 of banana also clustered into this group. The subgroups (I and II) are distinct with previous classification due to the analysis method and standard [7]. In the subgroup Ia, the selected monocots have at least two SROs except for banana, suggesting that duplication event in the lineage might happen after banana. Actually, the expansion event had happened in *P. patens*. There were three predicted SROs in subgroup Ib. Banana chromosome 4 and 5 contained two *MaSRO* genes, respectively. It is assumed that maintenance of duplicate genes provides the redundancy function in stress response and plant development, such as AtRCD1 and AtSRO1 [9].

The tissue-specific transcript analysis showed that *MaSRO1*, *3*, *4* and *6* were more abundant in stems and leaves, while *MaSRO2* was highly expressed in roots, like their counterparts in rice [20]. The high abundance of *MaSRO* expression in stems and leaves might participate in the regulation of plant growth and development. Candidates with high transcripts in roots may be important for perceiving various environmental stimuli. We also found that most of *MaSROs* in the group I were upregulated under PEG treatment (Fig. 4). Likewise, the mutated plants of rice *OsSRO1c* and *Arabidopsis rcd1–3*/*sro1–1* demonstrated the increase of stomatal aperture and sensitivity to drought [15, 20]. It seems that *SROs* might be involved in maintenance of cell turgor pressure and decrease of membrane injury under osmotic stress. In addition, qRT-PCR analysis showed that each *MaSROs* had at least one response to heat, UV cold, salt, and wounding treatments (Fig. 4). Therefore, different SROs may contribute to diverse abiotic responses. It was supported that the *rcd1–1* mutant was more tolerant to UV than the wild-type plants [14]. Compared to the function of SROs under abiotic stress, there is little knowledge of SROs in response to biotic stress until now. Previous study indicated that *AtRCD1* was involved in the regulation of plant growth, but not in the activation of defense response [29]. We found that more than half of *MaSROs* displayed differential expression in banana roots inoculated with *Foc* TR4. Pharmacological inhibition of the PARP protein blocks the plant response to biotic stress induced by microbe associate molecular patterns [30]. In *Arabidopsis,* loss of PARG1 (poly(ADP-ribose) glycohydrolase) causes greater susceptibility to the necrotrophic pathogen *Botrytis cinerea* [31, 32]. Hence, *MaSROs* own different regulation mechanisms under biotic and abiotic stress treatments.

Accumulated evidences indicated that various plant hormones are important for plant’s resistance responses to abiotic and biotic stresses [4–6, 13]. In the present study, most of *MaSROs* were upregulated by ABA, ethylene and GA treatments. But only *MaSRO3* and *MaSRO4* exhibited the induced expression under SA treatment (Fig. 5). The previous study showed that abiotic stress responses were largely controlled by ABA, and the priming SA could predominately induce defense against different biotic assailants [3]. It promotes us to speculate that *MaSRO3* and *MaSRO4* induced by multiple stresses might be involved not only in abiotic stress, but also in biotics stress. Indeed, gene expression depends on the presence of multiple CREs, which integrate signals from diverse TFs to control cell perception and response to environment factors [33]. The ABA response elements (ABREs) were one of the most common CREs in all *MaSRO* promoters. It is likely consistent with the fact that the transcript accumulations of *MaSROs* were generally induced by ABA treatment (Fig. 5). Most of the identified CREs within the *MaSRO4* promoter could account for its expression levels upregulated by numerous stresses and hormones. Similarly, *MaSRO2* are more responsive to ethylene and abiotic stresses, which might be related to the number and type of CREs. By contrast, relatively few CREs were found in the *MaSRO1* promoter, corresponding with low or no obvious expression after different treatments (Figs. 4 and 5). Overlapping CREs imply a complexity of gene regulatory network in response to different environmental stimuli. Both ABRE and C-repeat motif/drought-responsive element (CRT/DRE) were also detected in the promoters of cold-responsive genes [34, 35]. The ABRE serves as a coupling factor that functions cooperatively with the CRT/DRE in response to drought and high salinity [33]. Therefore, *MaSROs* might control a switch in priority between biotic and abiotic stresses by the regulation of a complex hormone network.

Based on transcript characteristics of *MaSRO4* induced by multiple stresses and hormone treatments, we analyzed the interaction partners of MaSRO4, which could directly interact with MaMYB4 and MaNAC6 (Fig. 8). Overexpression of a *MaMYB4* homolog, *OsMYB4*, effectively upregulated transcript levels of defense genes and improved stress resistance [36]. NAC was also considered as an interaction center between biotic and abiotic signaling pathways, activated by JA, ethylene, ABA, and SA [37, 38]. Likewise, AtRCD1 or OsSRO1c could interact with a number of TFs in responses to different stress treatments [7, 9]. Interestingly, the AP2 can interact with AtRCD1, AtSRO1 and OsSRO1c [7, 9], but no interaction with their homolog MaSRO4 was detected in the yeast-two-hybrid system. It suggests that functional diversity of the SRO family could be caused by the selective interaction with specific TFs.

To further identify the interaction domain of MaSRO4 with TFs, different N- and C-terminal truncations were generated. Analysis of conserved AAs supported that the WWE domain could form a half of a beta-barrel with a positively charged PAR-binding pocket [39]. However, the deletion of WWE domain did not affect the interaction of MaSRO5 with MaMYB4 (Fig. 8c), suggesting that the domain is not dispensable for plant’s survival. Although the conserved PARP, such as AtRCD1, does not possess ADP-ribosyl transferase activity in plants [7], the PARP domain of MaSRO5 was required for interacting with MaMYB4 (Fig. 8c). The partial or complete loss of the PARP domain within AtRCD1 and AtSRO1 results in a varied expression and a loss of stress tolerance [9, 15]. It is supported that the functional PARP domain of wheat *TaSRO1* could complement the resistance of *Arabidopsis* plants to salinity stress [21]. However, whether PARP provides allosteric regulation of plant SROs’ function still needs to be determined. Compared with the RST domain, the more conservation of amino-acids was found in the C-termini of monocot SROs (Additional file 6). It appears that there are conserved mechanisms of stress resistance in monocots. The RST domain of dicots and monocots likely owns different function in the recruitment of proteins that are involved in activating gene expression. For example, the RST domains of AtRCD1 and AtSRO1 were important for the interaction with TFs [7, 15, 16]. However, the RST domain of MaSRO4 was not necessary for interacting with MaMYB4 in our study (Fig. 8c). Likewise, OsSRO1c without the RST domain still interacts with OsDREB2B [20]. Therefore, these domains of SROs are crucial for the functional diversity and the activation of multiple signaling pathways. How different SROs specifically recognize critical TFs and regulate expression of downstream genes is still unknown. Analysis of motif function, by protein-interaction analyses of TF complexes, is needed.

## Conclusions

In our present study, six *MaSROs* were identified and characterized in banana genome. A total of 77 *SRO* members from 15 species were clustered into two main group with a distinct structure difference. The monocot *SROs* were first found in the group II. The qRT-PCR assay showed that half of *MaSROs* had high expression levels in banana stems and roots. Based on transcript levels induced by diverse stress and hormone treatments, *MaSROs* might participate in the crosstalk of complex stress signaling pathways. The numbers and types of CREs further support expression profiles of *MaSROs*. Especially, *MaSRO4* with numerous CREs in the promoter region exhibited positive responses to multiple stress treatments, suggesting that it could be a good source for breeding stress-tolerant cultivars. The yeast two-hybrid and Co-IP assays demonstrated that MaSRO4 could interact directly with MaNAC6 and MaMYB4 through the PARP domain. Therefore, our results provide some novel information to stress-related physiological functions of MaSROs, but their detailed roles still need a number of experimental validation.

## Methods

### Plant materials and growth conditions

Micropropagated banana plantlets of “Williams (Cavendish subgroup, AAA)” and *Foc* TR4 were obtained from the Chinese Academy of Tropical Agricultural Sciences, Haikou, China. Plantlets were cultured on the Murashige-Skoog medium [40]. Before treatment experiments of biotic and abiotic stresses, micropropagated plants were acclimatized in a secure shade environment as described by Wang et al. (2012) [41].

### Identification of SROs and phylogenetic tree construction

To identify SROs from 16 different species, sequence files were downloaded from the respective project databases, including banana [42, 43]: the Banana Genome Hub (http://banana-genome-hub.southgreen.fr/, DH Pahang Version 2), *Arabidopsis thaliana* [44]: the *Arabidopsis* Information Resource (http://www.arabidopsis.org), *Brachypodium distachyon* [45]: the Munich Information Center for Protein Sequences (http://mips.helmholtz-muenchen.de/plant/brachypodium, Version 1.2), *Medicago truncatula* [46]: the HapMap project (http://www.medicagohapmap.org), *Oryza sativa* [47]: the Rice Genome Annotation Project (http://rice.plantbiol ogy.msu.edu, Version 7.0), *Physcomitrella patens* [48]: the *Physcomitrella patens* Resource (http://cosmoss.org/, Version 3.3), *Zea mays* [49]: the Maize Genetics and Genomics Database (https://www.maizegdb.org/, Version 3.0), and *Malus domestica* [50]: the Genome Database for Rosaceae (http://www.rosaceae.org/). Two websites (http://www.phytozome.net and https://www.ncbi.nlm.nih.gov/) were used to search the SROs from *Glycine max* (Version 2.0) [51], *Solanum lycopersicum* [52], *Setaria italic* (Version 2.2) [53], *Populus trichocarpa* (Version 3.0) [54], *Vitis vinifera* [55], *Phoenix dactylifera* [56], *Elaeis guineensis* [57], and *Chlamydomonas reinhardtii* [58]. Moreover, the PARP (PF00644) and RST (PF12174) domains from the Pfam database (http://pfam.xfam.org/, Pfam 32.0 accessed in September 2018) were used as a query to identify all SRO sequences in the respective project databases (*p*-value = 0.001) [7]. The redundant sequences were removed by the decrease redundancy tool (http://web.expasy.org/decrease_redundancy). The candidate protein sequences were finally confirmed using ScanProsite (https://prosite.expasy.org/scanprosite/) [59] and SMART (http://smart.embl-heidelberg.de/, accessed on 4 January 2018) [60].

The full-length SRO protein sequences were aligned using a ClustalW program (MEGA version 7.0.1) with default parameters [61]. To compare the domain conservation of SRO evolution, the PARP domain sequences from SROs were also used to do the homology alignment. Two phylogenetic trees were constructed using the Maximum-Likelihood (ML) method of MEGA 7.0.1 with the following parameters: WAG protein substitution model, gamma distribution and bootstrap (1000 replicates). The molecular weight, isoelectric point and grand average of hydrophobicity (GRAVY) of each MaSRO were calculated using the ExPASy website (http://www.expasy.org/tools/) [62].

### Prediction of chromosomal location, gene structure and CREs of *MaSRO*s

The starting and ending positions of all *MaSROs* on each chromosome were obtained from the banana genome database. The exon-intron structure of each *MaSRO* was determined by aligning the full-length cDNA sequence with the genomic DNA sequence. The schematic structure of each *MaSRO* was drawn by the Gene Structure Display Server (http://gsds.cbi.pku.edu.cn/index.php, Version 2.0) [63]. The putative CREs were identified from the 1500 bp upstream sequences of the start codon of each *MaSRO* using the PlantCARE (http://bioinformatics.psb.ugent.be/webtools/plantcare/html/) [64].

### Prediction of conserved motifs and subcellular localizations of MaSROs

The conserved motifs were detected using a MEME tool (http://meme-suite.org/tools/meme, Version 5.0.4), following the optimum motif width (≧10 and ≦150) and seven motifs. The Plant-mPLoc server (http://www.csbio.sjtu.edu.cn /bioinf/plant-multi/, 2.0 version) was used to predict the subcellular localizations of MaSROs [65].

### Banana seedlings treated with various stresses and inoculated with *Foc* TR4

For osmotic and salt stresses, the banana roots of six-leaf seedlings were treated with 15% of PEG6000 and 200 mM of NaCl solutions [66], respectively. The samples were collected at 0, 3, 12 and 24 h after osmotic and salt stress treatments. For cold and heat shock stress, seedlings were transferred to a growth chamber at 4 °C (sampled at 0, 3, 12 and 24 h) and 42 °C (sampled at 0, 0.5, 2 and 4 h), respectively. For UV treatment, unfiltered germicidal emitting lamps (*λ* = 254 nm) (TUV 15 W/G15 T8, Phillips, Netherlands) was located 15 cm above the illumination area for 0, 3, 6, and 12 h. Banana root tips were wounded with a scalpel and put on water at room temperature for 0, 1, 3 and 6 h. For exogenous hormone treatments, banana roots were cultured in the nutrient solution added with 100 μM of ABA, SA, GA3 or ethylene, respectively. The treated roots were sampled after 0, 3, 6 and 12 h. For *Foc* TR4 inoculation, this strain was cultured on half strength potato dextrose agar at 25 °C for two weeks. The spores were then centrifuged at 5000 rpm for 5 min and the pellet was re-suspended in double distilled water. The optical density of fungal suspension was adjusted to 10^6^ spores per ml with sterile water. The roots were dipped into 15 ml of fungal suspension in a Petri dish (9 cm in diameter), and were sampled at 0, 2, 24, 48 and 72 h. All samples were rapidly frozen in liquid nitrogen for RNA isolation. Each experiment was repeated three times.

### RNA isolation and qRT-PCR

Total RNAs of different banana tissues were isolated by RNeasy Plant Mini Kit (Qiagen, Valencia, California, USA). The first strand of cDNAs was synthesized from 2 μg of DNaseI-treated total RNA using RevertAid™ First Strand cDNA Synthesis Kit (Thermo Scientific, USA) according to the manufacturer’s instruction. The LightCycler® 480 SYBR Green I Master Mix was used for qRT-PCR analysis in a LightCycler® 480 System (Roche Diagnositcs, Mannheim, Germany) according to the standard protocol. A total 10 μl of reaction solution contained 0.1 μl of reverse and forward primers (100 pmol), 1 μl of cDNA, 5 μl of FastStart SYBR Green I Master Mix and 3.5 μl of nuclease-free water. The PCR reaction was carried out as follows: 95 °C for 3 min and 40 cycles (95 °C for 10 s, 57 °C for 20 s and 72 °C for 20 s). The banana *18S rRNA* gene (GenBank ID: U42083) was used as an internal control. All reactions were repeated with triple biological replicates. These primer sequences were listed in Additional file 10.

### Yeast two-hybrid screening

The full-length or truncated *MaSRO5* was fused to the Gal4 DNA binding domain (BD) in pGBKT7, and was transformed into the yeast strain AH109. The transformants were grown on SD/−Trp plates. The lacZ assay was performed to examine self-activation of the full-length or truncated *MaSRO5*. The identified yeast strain AH109 harboring the Gal4-BD -*MaSRO5* fusion was mated with Y187 (MATα) containing the Gal4-AD-TF in pGADT7 (GAL4 AD fusion) on 96-well plates. The mating products were plated onto SD/−Trp/−Leu and SD/−Trp/−Leu/−His/−Ade supplemented with 3-amino-1,4,5-triazole (3-AT). The yeast growth was followed for up to 7 d, and positive colonies were subjected to *α*-galactosidase assay on SD/−Trp/−Leu/−His/−Ade/X-α-Gal. Plates were incubated at 30 °C and photographed after 5 d of growth.

### Co-immunoprecipitation assay

Arabidopsis protoplasts were prepared from 4-week-old plants according to the protocol described by Yoo et al. (2007) [67]. *MaSRO5*-HA was co-expressed with TF-FLAG in Arabidopsis protoplasts. For protein expression, protoplasts (1 ml, 2 × 10^6^ cells) were transfected with 100 μg of plasmid DNA. Transfected protoplasts were incubated in the dark at room temperature for 16 h. The proteins were purified using commercial affinity resins (anti-FLAG M2-resin from Sigma and anti-HA 3F10 resin from Roche). The immuno-precipitates were separated by 10% SDS-PAGE and proteins on immunoblots were detected with anti-FLAG and anti-HA antibodies [68].

### Statistical analyses

Data processing and statistical analysis were performed with the SPSS statistical software package (SPSS Inc., Cary, NC, USA, v.22). Differences between two treatments were statistically analyzed by the Student’s t test.

## Additional files

Additional file 1: The components of amino acids in the conserved motifs. (XLSX 10 kb)

Additional file 2: Chromosomal location, subcellular localization and putatively physicochemical characters of banana MaSROs. (XLSX 10 kb)

Additional file 3: Information of the SRO orthologs in representative sequenced 16 plant species. (XLSX 14 kb)

Additional file 4: Protein sequences used for the construction of phylogenetic trees. (TXT 39 kb)

Additional file 5: A newick file of the phylogenetic tree. (TXT 2 kb)

Additional file 6: Sequence logos of the WWE, PARP, and RST domains in dicots and monocots. The overall height of the stack represented the level of sequence conservation. Heights of residues within a stack indicated the frequency of each residue at the indicated position. (TIF 7444 kb)

Additional file 7: Distribution of conserved motifs in the SROs. The conserved motifs were identified through the MEME tool. The different colored boxes represented seven motifs. The scale at the bottom was used to estimate the lengths of proteins and motifs location of each motif. (TIF 2906 kb)

Additional file 8: Unrooted phylogenetic tree of the PARP domain of SROs from 15 plant species. The amino acid sequences of the PARP domain were aligned using Clustal W, and the phylogenetic tree was constructed using MEGA 7.0. (TIF 947 kb)

Additional file 9: Expression analysis of six *MaSROs* in banana different tissues by qRT-PCR. Total RNAs were isolated from the roots, stems and leaves of the six-leaf banana seedlings and fruits, respectively. Data indicated relative expression levels (means ± SE) from three independent biological replica (three RNA extractions; *n* = 3). Used primers of *MaSROs* were listed in Additional file 10. (TIF 1464 kb)

Additional file 10: The primer sequences were used in the present study. (XLSX 11 kb)

## Abbreviations

ABA: Abscisic acid
ABREs: ABA response elements
Co-IP: Immunoprecipitation
CRT/DRE: C-repeat motif or drought-responsive element
*Foc* TR4: *Fusarium oxysporum* f. sp. *cubense* tropical race 4
hpi: Hour post inoculation
JA: Jasmonic acid
PARP: Poly(ADP-ribose) polymerase
PEG: Polyethylene glycol
qRT-PCR: Quantitative real-time polymerase chain reaction
ROS: Reactive oxygen species
RST: RCD1-SRO-TAF4
SA: Salicylic acid
SRO: SIMILAR TO RCD ONE
TF: Transcription factor
UV: Ultraviolet
