# Supplementary figures and images for: Genome-wide characterization of a SRO gene family involved in response to biotic and abiotic stresses in banana (Musa spp.)
Source: BMC Plant Biol. 2019 May 22;19:211. doi: 10.1186/s12870-019-1807-x (PMC6530135; doi:10.1186/s12870-019-1807-x)

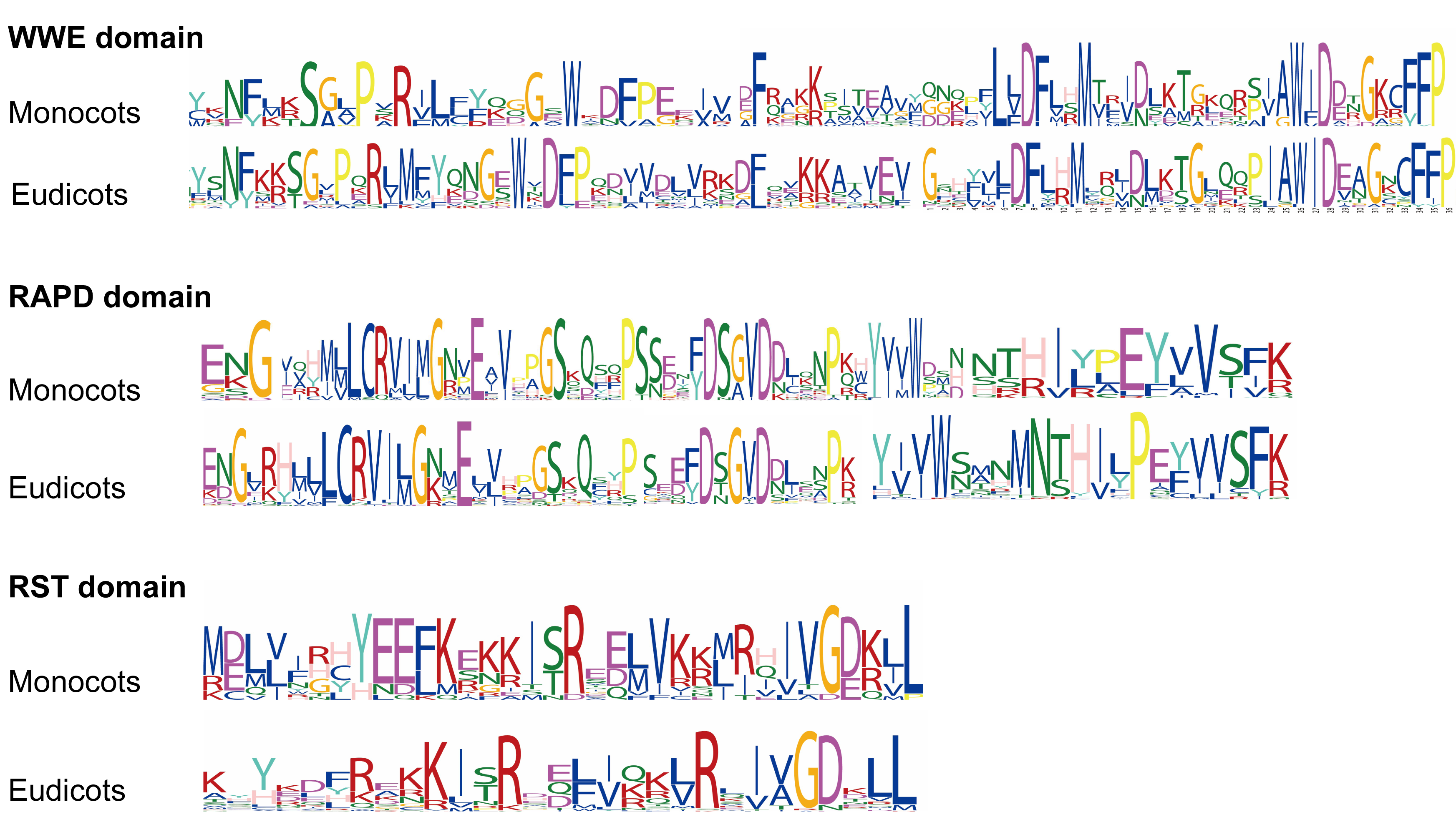

Supplement: Supplementary file 6 — Sequence logos of the WWE, PARP, and RST domains in dicots and monocots. The overall height of the stack represented the level of sequence conservation. Heights of residues within a stack indicated the frequency of each residue at the indicated position. (TIF 7444 kb) [file 12870_2019_1807_MOESM6_ESM.tif]

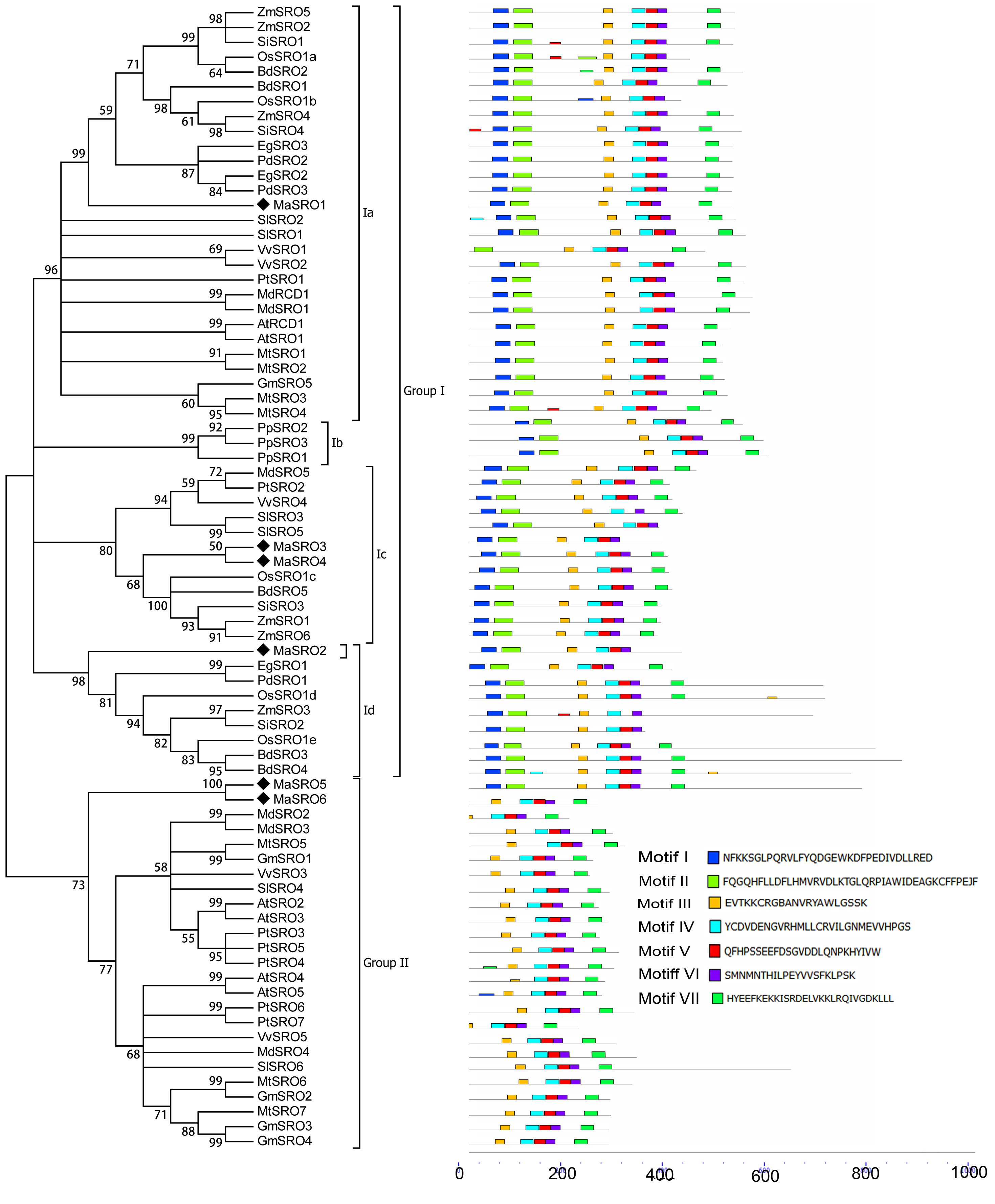

Supplement: Supplementary file 7 — Distribution of conserved motifs in the SROs. The conserved motifs were identified through the MEME tool. The different colored boxes represented seven motifs. The scale at the bottom was used to estimate the lengths of proteins and motifs location of each motif. (TIF 2906 kb) [file 12870_2019_1807_MOESM7_ESM.tif]

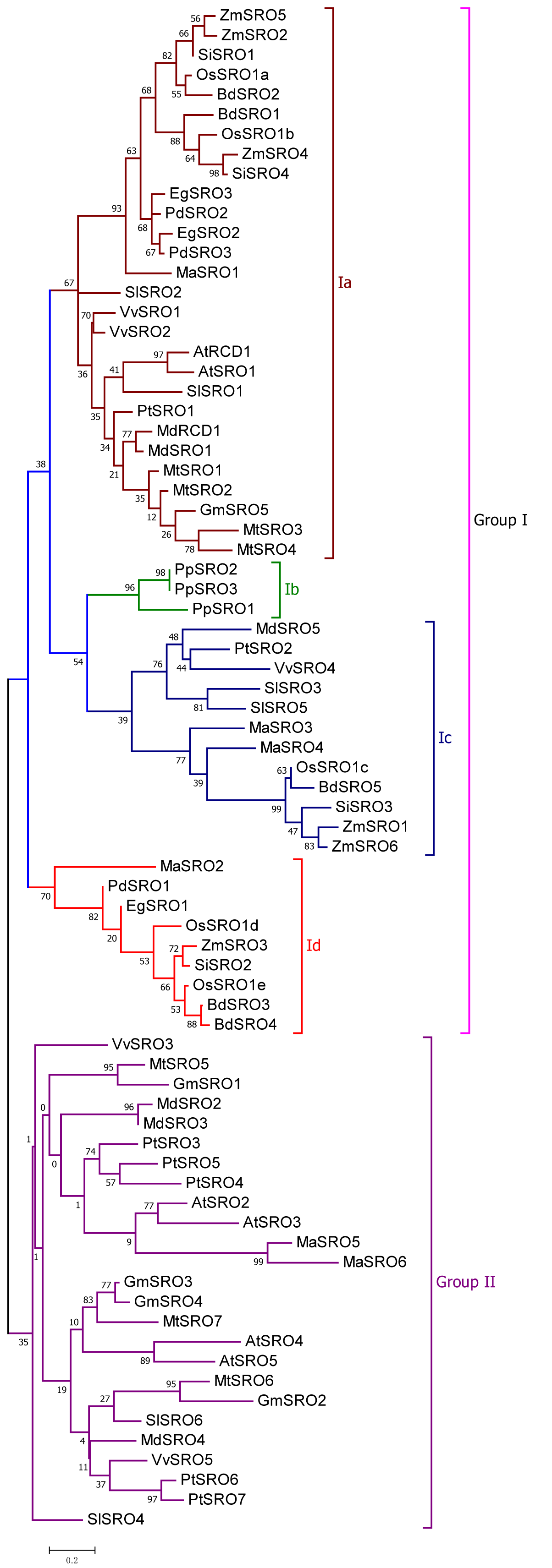

Supplement: Supplementary file 8 — Unrooted phylogenetic tree of the PARP domain of SROs from 15 plant species. The amino acid sequences of the PARP domain were aligned using Clustal W, and the phylogenetic tree was constructed using MEGA 7.0. (TIF 947 kb) [file 12870_2019_1807_MOESM8_ESM.tif]

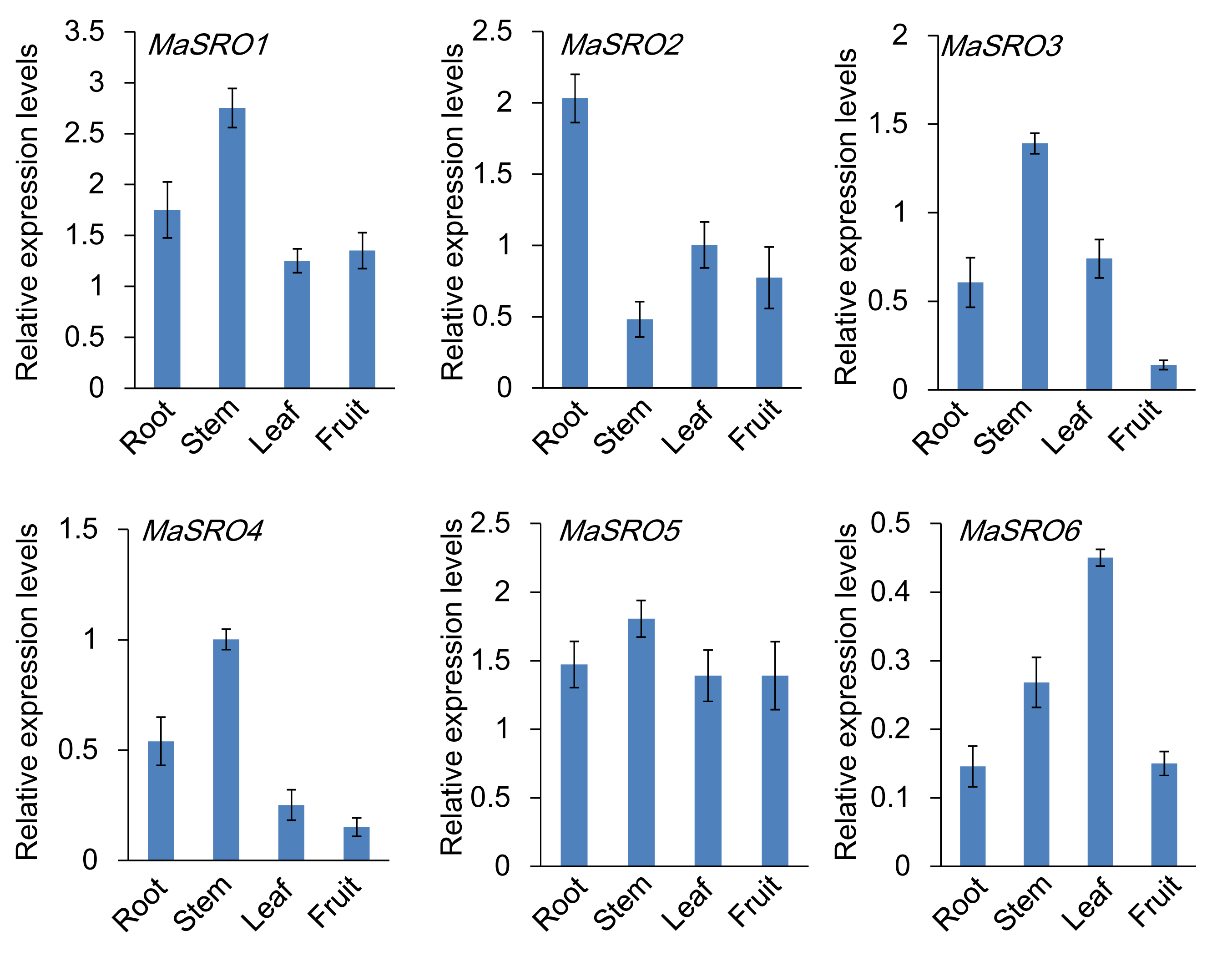

Supplement: Supplementary file 9 — Expression analysis of six MaSROs in banana different tissues by qRT-PCR. Total RNAs were isolated from the roots, stems and leaves of the six-leaf banana seedlings and fruits, respectively. Data indicated relative expression levels (means ± SE) from three independent biological replica (three RNA extractions; n = 3). Used primers of MaSROs were listed in Additional file 10. (TIF 1464 kb) [file 12870_2019_1807_MOESM9_ESM.tif]
